# AI-Driven Innovations in Neuroradiology and Neurosurgery: Scoping Review of Current Evidence and Future Directions

**DOI:** 10.3390/cancers17162625

**Published:** 2025-08-11

**Authors:** Bartosz Szmyd, Małgorzata Podstawka, Karol Wiśniewski, Karol Zaczkowski, Tomasz Puzio, Arkadiusz Tomczyk, Adam Wojciechowski, Dariusz J. Jaskólski, Ernest J. Bobeff

**Affiliations:** 1 Department of Neurosurgery and Neuro-Oncology, Medical University of Lodz, Barlicki University Hospital, Kopcinskiego St. 22, 90-153 Lodz, Poland; bartosz.szmyd@umed.lodz.pl (B.S.); malgorzata.podstawka@umed.lodz.pl (M.P.); karol.wisniewski@umed.lodz.pl (K.W.); karol.zaczkowski@umed.lodz.pl (K.Z.); dariusz.jaskolski@umed.lodz.pl (D.J.J.); 2 Department of Pediatrics, Oncology and Hematology, Medical University of Lodz, Sporna St. 36/50, 91-738 Lodz, Poland; 3 Department of Diagnostic Imaging, Polish Mothers’ Memorial Hospital Research Institute, 93-338 Lodz, Poland; tomasz.puzio@iczmp.edu.pl; 4 Institute of Information Technology, Faculty of Technical Physics, Information Technology and Applied Mathematics, Lodz University of Technology, al. Politechniki 8, 93-590 Lodz, Poland; arkadiusz.tomczyk@p.lodz.pl (A.T.); adam.wojciechowski@p.lodz.pl (A.W.); 5 Department of Sleep Medicine and Metabolic Disorders, Medical University of Lodz, Mazowiecka St. 6/8, 92-251 Lodz, Poland

**Keywords:** neuroradiology, neurosurgery, artificial intelligence, AI, glioma, IDH

## Abstract

**Background/Objectives**: The rapid development of artificial intelligence is transforming the face of medicine. Due to the large number of imaging studies (pre-, intra-, and postoperative) combined with histopathological and molecular findings, its impact may be particularly significant in neurosurgery. We aimed to perform a scoping review of recent applications of deep learning in MRI-based diagnostics of brain tumors relevant to neurosurgical practice. **Methods**: We conducted a systematic search of scientific articles available in the PubMed database. The search was performed on 22 April 2024, using the following query: ((MRI) AND (brain tumor)) AND (deep learning). We included original studies that applied deep-learning methods to brain tumor diagnostics using MRI, with potential relevance to neuroradiology or neurosurgery. A total of 893 records were retrieved, and after title/abstract screening and full-text assessment by two independent reviewers, 229 studies met the inclusion criteria. The study was not registered and received no external funding. **Results**: Most included articles were published after 1 January 2022. The studies primarily focused on developing models to differentiate between specific CNS tumors. With improved radiological analysis, deep-learning technologies can support surgical planning through enhanced visualization of cerebral vessels, white matter tracts, and functional brain areas. Over half of the papers (52%) focused on gliomas, particularly their detection, grading, and molecular characterization. **Conclusions**: Recent advancements in artificial intelligence methods have enabled differentiation between normal and abnormal CNS imaging, identification of various pathological entities, and, in some cases, precise tumor classification and molecular profiling. These tools show promise in supporting both diagnosis and treatment planning in neurosurgery.

## Simple Summary

The rapid development of artificial intelligence is transforming the face of medicine. Due to the large number of imaging studies (pre-, intra-, and postoperative) combined with histopathological and molecular findings, its impact may be particularly significant in neurosurgery. Our scoping review showed that recent advancements in artificial intelligence methods have begun to enable differentiation between normal and abnormal central nervous system (CNS) imaging findings, distinguishing various pathological entities, and, in some cases, even precise tumor classification, identification of tumor molecular background, and planning radiotherapy.

## 1. Introduction

Brain pathologies are classified according to various criteria, such as etiology, localization, pathophysiological mechanisms, and clinical presentation. In clinical practice, the main categories include neurodegenerative disorders, vascular diseases, brain tumors, developmental disorders, and post-traumatic lesions. The incidence of brain tumors constitutes a significant public health issue, with a noted increase in the occurrence of benign central nervous system (CNS) tumors [1]. Aneurysms and other vascular malformations are often detected incidentally in otherwise healthy patients and require intervention in selected cases [2]. With the increased use of imaging studies, there is a growing need for a better understanding of the natural history of these lesions and improved strategies for early detection and management.

In recent decades, advancements in diagnostic technologies have significantly improved the diagnosis and treatment of brain diseases. Imaging diagnostics often start with a non-contrast computed tomography (CT) scan of the head, as it is a rapid and widely accessible method that provides an initial assessment of pathological lesions, mass effects, and indications for urgent neurosurgical intervention. Contrast-enhanced CT is particularly useful when MRI is contraindicated, for example, due to metallic implants in the patient. However, as a method utilizing ionizing radiation, CT carries a certain radiation risk.

Brain MRI is a non-invasive examination and allows precise evaluation of soft tissues, making it the gold standard in brain disease diagnostics. Contrast-enhanced MRI enables accurate assessment of the location, character, and position of lesions in relation to the meninges, intracranial vessels, and the ventricular system. MRI is versatile, allowing customization of sequences based on the type of lesion evaluated; for example, T1- and T2-weighted sequences assess morphological structures of the brain, FLAIR sequences detect brain edema, and DWI sequences identify areas of ischemia. Contrast-enhanced T1-weighted imaging is considered the most universal MRI sequence, often referred to as the “pathological sequence,” as contrast enhancement highlights intracranial vessels and hypervascular pathological areas. This facilitates detailed evaluation of both benign and malignant brain tumors, as well as intracranial aneurysms and other vascular malformations, enhancing diagnostic effectiveness and treatment planning.

Additional imaging modalities used in clinical practice include CT angiography (CTA), conventional angiography, and MR angiography (MRA). CTA enables exclusion of vascular malformations and assessment of intracranial aneurysm morphology. Conventional angiography is considered the most accurate method for assessing cerebral vascular anomalies and additionally allows for embolization of skull base tumors prior to neurosurgical interventions. It is a dynamic study, assessing arterial, capillary, and venous phases, crucial for diagnosing arteriovenous malformations. MRA is a non-invasive examination applied in monitoring intracranial aneurysms that are under surveillance.

Radiologists interpret imaging studies to provide diagnostic insights that guide clinical decision-making. However, there are no universal standards or templates for these reports; hence, each one is unique, and different radiologists may describe the same findings in different ways. In recent years, the growing availability of CT and MRI scanners has led to more imaging studies being performed, but without a matching increase in the number of radiologists. As a result, waiting times for reports have lengthened. Some private diagnostic facilities even offer MRI scans without interpretation by a radiologist, which many physicians view as unethical and potentially harmful to patients. The increased demand for radiological reporting results from technological advances, rising demand for imaging diagnostics, and expanding medical applications of these technologies. According to the Naczelna Izba Kontroli (Polish Supreme Audit Office), recent years have seen substantial growth in medical diagnostics through MRI, but there remains a need to address the suboptimal use of this advanced technology, partly due to a shortage of radiology specialists and ineffective procurement planning [3]. Problems such as the growing shortage of radiologists, increased numbers of diagnostic examinations, overuse of low-value procedures, and resistance to implementing clinical decision support software are observed in Poland and worldwide [4]. Addressing these challenges requires ensuring adequate quality and accessibility of imaging diagnostics, crucial for patient safety.

The use of deep-learning neural networks (DLNN) in combination with MRI opens new possibilities for identifying brain diseases [5]. Three main machine-learning strategies used in medicine are supervised learning, unsupervised learning, and reinforcement learning. In our recent study, we employed supervised learning to develop a DLNN model analyzing non-contrast head CT scans from emergency departments, enabling automated segmentation of intracranial compartments and cerebrospinal fluid, which allowed quantitative evaluation of mass effect and identified patients requiring neurosurgical intervention [6].

Integration of DLNN models with brain MRI has the potential to revolutionize neurological diagnostics by accelerating patient triage, supporting histopathological diagnostics of brain tumors, and improving the detection and morphological assessment of intracranial aneurysms. Recent studies have demonstrated the utility of generative adversarial networks for enhancing brain tumor classification by generating synthetic MRI datasets, thereby addressing data scarcity and privacy concerns while achieving high diagnostic accuracy [7,8]. The primary advantages of DLNN models include precise volumetric assessment and reproducibility, essential for monitoring pathological changes. Reports generated by DLNN models function independently of human factors such as fatigue or time of day, ensuring consistent quality and objectivity. Additionally, these standardized and repeatable reports can provide valuable tools for comparative analyses across various clinical cases. By providing radiologists with quantitative volumetric measurements and preliminary qualitative assessments, DLNN models combined with MRI have the potential to standardize intracranial lesion reporting and reduce reporting turnaround times.

In the current paper, we aimed to perform a scoping review of recent applications of deep learning in MRI-based diagnostics of brain tumors relevant to neurosurgical practice.

## 2. Materials and Methods

**Figure 1:**
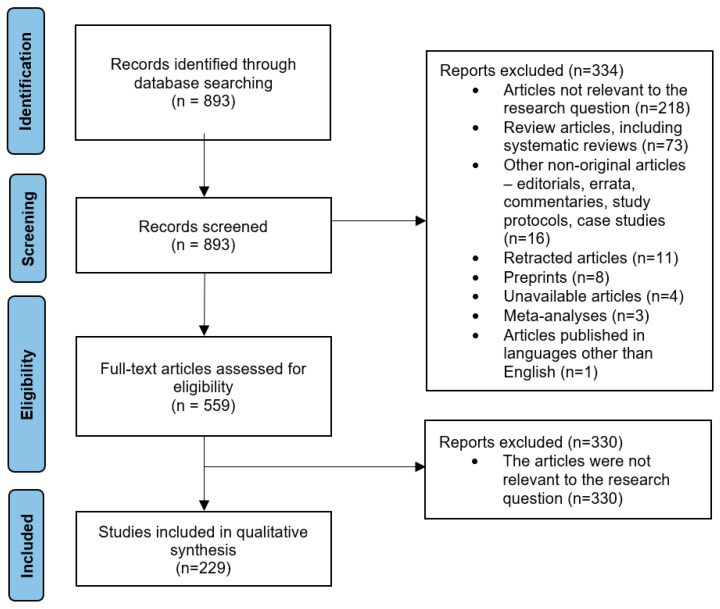
Flowchart of article selection for the study according to PRISMA guidelines.

To achieve a comprehensive understanding of the current state of knowledge, we conducted a systematic review of scientific articles available in the PubMed database. The articles were searched on 22 April 2024, using the following query: ((MRI) AND (brain tumor)) AND (deep learning). From the retrieved 893 records, 559 were assessed according to the original character of the study, showing potential diagnostic usage in neuroradiology/neurosurgery (see Figure 1). Finally, 229 articles were included for analysis. All decisions were made independently by two authors based on the following inclusion and exclusion criteria:

Inclusion criteria:

- Original research article
- Use of artificial intelligence modality
- Application to currently available radiological modalities
- Potential relevance to clinical workflows in neuroradiology or neurosurgery (e.g., classification, segmentation, molecular prediction)

Articles were excluded if they were:

- Reviews, editorials, conference abstracts, or letters
- Not related to neuroradiology or neurosurgery

While inter-rater agreement was not formally quantified (e.g., via Cohen’s kappa), high concordance was achieved during the screening process. In cases of discrepancies, the senior author was consulted for clarification. This review was performed in accordance with the PRISMA (Preferred Reporting Items for Systematic Reviews and Meta-Analyses) guidelines for scoping reviews (namely: PRISMA Extension for Scoping Reviews). This review was not registered, as PROSPERO does not accept registrations of scoping reviews.

This review was conducted with the aim of mapping the scope and thematic distribution of recent research on deep-learning applications in brain tumor imaging. As such, we did not define specific clinical outcomes or assess risk of bias using formal tools. Our data extraction focused on the types of tumors studied, analytical goals, and deep-learning approaches used. The review does not include pooled effect estimates or comparative efficacy data, and therefore, no outcome filtering, bias assessment, or imputation methods were applied.

Regarding synthesis methods, given the exploratory and descriptive nature of this review, no quantitative synthesis or meta-analysis was performed. The included studies were selected for narrative synthesis based on thematic relevance to deep-learning applications in MRI-based brain tumor diagnostics. No data conversions, imputations, or statistical models were applied. Key study characteristics were extracted and presented in tabular form. Due to the heterogeneity of study designs and absence of standardized outcomes, no formal subgroup analysis, heterogeneity assessment, or sensitivity analyses were conducted. Finally, no formal assessment of reporting bias was conducted, as no quantitative synthesis or pooled outcome analysis was performed in this review.

## 3. Results

Most articles were published after 1 January 2022. Within the structure of retrieved articles, particular attention is drawn not only to the exponential growth in new studies but also to the relatively small proportion of non-original articles (n = 92; 10.3%). This translates into an increasing number of preprints (n = 8; 0.9%) and a relatively high proportion of retracted papers (n = 11; 1.2%). This situation highlights an even greater need for high-quality research in this area.

Of the 330 articles rejected at the full-text verification stage, many focused on basic applications of advanced technologies in neuroradiology, particularly artifact minimization [9,10], distinguishing normal images from pathological changes [11,12], or solely segmenting these changes [13,14]. Selected articles emphasized comparisons between different techniques, including not only machine learning but also general artificial intelligence approaches.

One of the main questions posed to artificial intelligence models in neurosurgery is the differentiation between specific types of lesions (see Table 1). This was clearly reflected in our review. Articles included in the review attempted to develop solutions for differentiating specific CNS tumors, such as glioblastoma, from solitary metastatic tumors [15,16,17,18,19,20,21,22,23]. Other tools aimed to differentiate gliomas from lymphomas [24] or meningiomas [25,26]. Some solutions expanded diagnostic capabilities, encompassing lesions such as (1) glioblastoma, solitary metastases, or CNS lymphomas [27,28]; (2) gliomas, pituitary tumors (without further specification), or meningiomas [29,30,31,32,33,34,35,36]; (3) ependymomas, meningiomas, medulloblastomas [37]; and (4) high-grade pediatric gliomas, medulloblastomas, and other tumors disseminating via cerebrospinal fluid [38]. Broader panels included (1) low- and high-grade gliomas, CNS metastases, meningiomas, pituitary adenomas, and vestibular schwannomas [39]; or (2) high-grade gliomas, anaplastic gliomas, meningiomas, primary CNS lymphomas, and metastatic tumors [40]. Other studies focused on narrower diagnostic groups, differentiating between (1) hemangioblastomas and other cerebellar/brainstem tumors [30], (2) schwannoma-like lesions versus glioblastomas [41], or (3) gliomas versus germinomas [42]. Other solutions assessed tumor malignancy grades [43] or survival prediction [44,45,46,47].

Recognizing and classifying the lesion is a focused topic; subsequent solutions have aimed to support the next levels of the diagnostic–therapeutic pathway—particularly in surgical planning. With improved analysis of radiological studies, advanced technologies in neurology can enhance surgical planning through better imaging of cerebral vessels [48], white matter tracts [49,50,51,52], or functional brain mapping [53].

### 3.1. Gliomas

Gliomas are the most common intra-axial tumors of the CNS. In the therapeutic process, determining the tumor grade is of critical importance, as it is strongly influenced by specific molecular alterations. This emphasis on molecular profiling was reflected in the structure of the studies included in our systematic review. A significant portion of articles (n = 119, 52%) focused on glioma tumors (see Table 1). Of these, 17 (14%) addressed glioma detection and grading. Most articles (n = 55, 46%) attempted detailed molecular assessments, ranging from single parameters such as IDH mutation status [54,55,56,57,58,59,60,61,62,63,64,65,66,67,68,69], 1p/19q codeletion [70,71,72], and MGMT methylation status [73,74,75,76,77,78,79,80,81,82,83,84,85,86,87], to mutations in CDKN2A [88] and histone protein H3 K27M [89,90]. More complex tools enabled simultaneous assessment of several parameters, particularly IDH status combined with (1) 1p/19q codeletion [91,92,93], (2) 1p/19q codeletion and MGMT methylation status [94], (3) TERT mutations [95], (4) CDKN2A/B mutations [96], or finally (5) ATRX mutations, chromosome 7 and 10 aneuploidy, and CDKN2 mutations [97]. Numerous studies aimed at determining glioma molecular subtypes [98,99,100,101,102,103,104], including those based on RNA sequencing (RNAseq) clustering analyses [105]. Other glioma-related topics covered tumor recurrence/progression detection [106,107], comprehensive radiotherapy planning [108,109], or distinguishing between pseudoprogression and true progression/recurrence [110,111,112,113,114]. The last group of glioma studies focused on survival prediction [115,116,117,118,119,120,121,122,123], with some authors combining these topics [124,125,126,127,128].

### 3.2. Metastases

Among 32 articles on metastases, 15 (47%) targeted lesion detection and segmentation [129,130,131,132,133]. Individual articles attempted to identify primary tumor sites [134,135]. Some studies aimed to support radiotherapy planning or monitor lesions during radiotherapy [136,137,138,139,140]. Most attention among specific metastatic tumors was devoted to lung cancer metastases, including differentiation between small-cell and non-small-cell lung cancer metastases or survival prediction in these patient groups [141,142,143,144,145,146,147,148].

### 3.3. Others

Several studies focused on selected issues related to sellar region tumors. Ishimoto et al. evaluated deep learning for perioperative assessment of pituitary adenomas [149]. Subsequent studies focused on segmentation and classification [150,151], tumor grading [152], assessment of invasiveness, particularly cavernous sinus infiltration [153,154,155], or completeness of resection [156].

Interestingly, only a small proportion of the studies focused on the most common intracranial tumors—meningiomas—which encompass 15 distinct histopathological diagnoses. Few studies developed tools for identifying critical anatomical structures near meningiomas [157] or graded lesions [158,159]. One article assessed NF2 mutations and S100 protein expression via preoperative imaging [160]. Early suspicion of neurofibromatosis type 2 could facilitate early detection and effective treatment of other common CNS tumors in these patients, such as vestibular schwannomas, additional meningiomas, astrocytomas, or ependymomas.

The remaining single studies covered topics related to CNS lymphomas [161], posterior fossa tumors [162], ependymomas [163], or vestibular schwannomas [164,165,166,167].

The analysis of published articles highlights significant limitations. Many studies relied on databases with diagnoses based on previous CNS tumor classifications. The year 2021 introduced a profoundly revised classification deeply rooted in modern molecular research. The definition of glioblastoma was revised to differentiate it from grade-4 gliomas harboring IDH1/IDH2 mutations. Some solutions attempted differentiation among lesions relatively rarely challenging for neuroradiologists or neurosurgeons, such as schwannoma vs. glioblastoma vs. non-neoplastic lesions [41]. Other studies utilized repositories containing highly selected, exemplary imaging cases [37]. This approach considerably limits practical application in daily neurosurgical and neuroradiological practice, underscoring the need for extensive datasets with histopathologically confirmed diagnoses aligned with the latest WHO classification. Databases ideally should include volumetric contrast-enhanced T1 imaging [39,42,131,168].

## 4. Discussion

We are approaching a technological shift that may transform prevention, diagnosis, treatment, and patient monitoring. In neurosurgery, a key challenge is how to collect data that can reliably train AI systems, as these tools could directly influence surgical precision and outcomes.

The paradigm shift in neurosurgical oncology from a two-dimensional to a three-dimensional perspective can be understood on two levels. First, the traditional two-dimensional approach relates to the surgical concept of gross total resection (GTR) versus subtotal resection [169,170]. In neurosurgical practice, GTR refers to the complete macroscopic removal of a tumor; however, unlike in oncological surgery, it does not guarantee margin-negative resection [169,170]. Residual microscopic tumor cells often remain in adjacent brain structures that cannot be safely resected without risking neurological damage. A similar principle applies to benign tumors, which, due to their slower growth, are more often associated with so-called “recurrence”—though in many cases, this is better described as the progression of residual tumor cells rather than true recurrence. Ultimately, the probability of incomplete resection is typically higher than the likelihood of de novo neoplastic transformation [169,170].

The second aspect of this shift pertains to the way we interpret radiological imaging, which has historically relied on two-dimensional slices. With the advent of DLNN, it is now possible to analyze tumor volume and spatial characteristics with far greater precision [171]. Studies have shown that residual tumor volume after resection is a more accurate predictor of prognosis than either the preoperative tumor size or the percentage resected. It also correlates with the risk of recurrence and the likelihood of compression of surrounding brain structures [172,173]. In this context, a three-dimensional approach, enabled by DLNN-based volumetric analysis, offers an objective, reproducible framework for evaluating surgical outcomes and guiding further treatment [174,175].

Since the presence of “informational noise” significantly limits their clinical applicability [176], the most reasonable path forward appears to be supervised learning—teaching the model what we already know in order to obtain consistent, reliable outputs grounded in verified medical knowledge. In neurosurgery, particular attention is given to tools designed for interpreting imaging studies. Different researchers adopt different strategies to address this challenge. The approach that offers the greatest level of control over outcomes involves image segmentation and training convolutional neural networks (CNNs) to perform similar segmentations on other scans [177]. Many authors also employ a simpler, whole image classification strategy—essentially reducing the task to a binary decision: “disease present” or “disease absent.” While more straightforward, this method carries significant risks. By labeling an entire scan with a single outcome and asking the network to learn this mapping, we run the bigger risk of creating a “black box” system—where decisions are made in an unexpected way without transparency or interpretability [177].

The learning process of DLNN should, in many ways, resemble a structured curriculum—beginning with the recognition of anatomical patterns that are consistent across all patients. In our experience, human-in-the-loop (HITL) workflows have proven highly effective for this purpose [177]. We start with basic anatomical segmentation and gradually expand it by incorporating additional structural details. Once the model has developed a solid understanding of normal anatomy, we can begin introducing pathological changes and task it with classifying abnormalities. Anatomical structures are present in the vast majority of scans; even in the presence of pathology, they typically remain visible, albeit potentially distorted. This General-to-Specific learning approach closely mirrors the way humans learn during formal education and medical training.

Our literature review reveals a contrasting trend present in the majority of current studies. Most research focuses on solving highly specific clinical problems, such as predicting molecular lesions in glioma [54,55,56,57,58,59,60,61,62,63,64,65,66,67,68,69,70,71,72,73,74,75,76,77,78,79,80,81,82,83,84,85,86,87,88,89,90,91,92,93,94,95,96,97,98,99,100,101,102,103,104,105]. In our view, while such narrowly focused models may be appealing from a publication or academic perspective, they often provide limited value in routine hospital workflows and are unlikely to gain widespread clinical adoption. Based on our observations, human specialists still tend to outperform AI in complex or atypical cases—particularly when the model has been trained to distinguish only between two specific pathologies. When confronted with a third, similarly appearing lesion, such models are prone to misclassification by forcing the input into one of the known categories. This limitation is especially relevant in neuro-oncology, where tumors frequently vary in location, morphology, and stage. Consider, for example, ring-enhancing lesions and solid lesions with dural attachment; while these are most commonly glioblastomas and meningiomas, respectively, the differential diagnosis is wide and often nuanced [178,179]. In this context, it is far more valuable for a neural network to detect the presence of a pathology, even without precise classification, as this alone can serve as a “red flag” to prompt expedited radiological assessment. Of course, the model may suggest a suspected diagnosis such as lymphoma or metastasis, but the mere identification of a suspicious lesion of a certain volume is already extremely helpful in daily clinical workflows, especially for early detection and screening. Moreover, in cases where a lesion or residual tumor is already known and under monitoring, the most clinically relevant metric becomes volume. Tracking changes in tumor volume over time is crucial, and this is precisely where DLNNs can make a meaningful and immediate contribution to neuro-oncology [180].

There is a prevailing belief that combining multiple specialized models will eventually lead to a unified “one-model” solution capable of holistic interpretation. While this is certainly possible, it appears to require significantly greater computational resources that are not always available in a hospital setting. A hierarchical arrangement of specialized models is one potential strategy, in which each model independently evaluates whether a given input falls within its domain, essentially estimating the likelihood that the case belongs to its predefined category. In this setup, multiple models operate in parallel. This represents a Specific-to-General approach, building broader generalizations from task-specific models, which differs from the way humans typically learn (see Table 2). It is more akin to a multidisciplinary case discussion, where specialists from various fields evaluate complex cases collaboratively. It is an interesting and potentially powerful strategy, but one that demands the development and maintenance of a large number of “narrow” DLNN models. In theory, such a solution is feasible. However, if we could instead design a single, more comprehensive model that has been exposed to a wide range of data and pathologies from the outset, there is a strong possibility that it would not only offer comparable interpretative capabilities but also operate more efficiently.

A crucial issue is whether current models are trained in a controlled manner. Are we teaching the model our knowledge and expecting it to generalize it to new cases? Or are we allowing it to detect patterns we cannot see, and then relying on it to make decisions based on unknown mechanisms? In the latter case, the question arises: what exactly is the model basing its conclusions on, if not our existing medical knowledge?

Ultimately, the primary goal in medicine is patient benefit—extending life and improving its quality. Yet these endpoints are rarely addressed in AI-based medical research. If we do not clearly define our clinical objectives and, instead, allow models to learn whatever patterns they deem important, we risk building systems that are effective only in a statistical or technical sense—not in a way that truly serves patients.

A common AI pitfall illustrates this risk: a model is trained to distinguish between male and female faces. If most men in the dataset have short hair and most women have long hair, the model may rely solely on hair length to make its determination. While technically correct within that dataset, the model’s reasoning is clinically meaningless—and potentially misleading. Similar logic traps may occur in medical AI if we do not impose appropriate constraints and interpretability standards.

All of the above issues are directly addressed by current trends in AI. The HITL approach is closely related to a few intensively investigated learning paradigms:

- Active learning, where the additional training data can be provided by domain experts if they or the system itself detects such a need [181,182].
- Incremental (continual) learning, where the model enhances its knowledge progressively, avoiding forgetting about previously acquired information [183,184].
- Multi-task learning, where solving different but related tasks helps the model not only to discover common patterns, but also to exploit similarities and differences between tasks (transfer learning) [185,186].

In all of those cases, domain experts (surgeons, radiologists) can precisely plan training strategies based on their medical experience, in particular on the type of training they were subjected to.

There are numerous approaches that utilize deep-learning neural networks (DLNNs) for the analysis of MRI studies. The most commonly adopted solutions typically incorporate convolutional layers (CNNs), which to some extent emulate the functioning of the human visual cortex by using hierarchical filters to progressively extract increasingly complex spatial features. Additionally, attention mechanisms, originally introduced in transformer architectures that have proven highly successful in natural language processing, are also employed. In the context of image analysis, these attention-based models, vision transformers (ViTs), treat small patches extracted from the image as spatially arranged tokens, analogous to words in a sentence, thereby enabling a contextual understanding of visual content.

The utility of deep neural networks stems from their ability to learn semantically meaningful and spatially localized representations of image content (feature extraction) during the training process. These learned representations are subsequently processed by dedicated decoding modules, depending on the specific downstream tasks. Notably, for performance and memory efficiency reasons, these intermediate representations are typically downsampled, i.e., stored at reduced spatial resolution.

When the target task involves predicting global properties of objects within an image (i.e., making predictions based on the entire image), the encoded feature structure is usually flattened, followed by the use of fully connected layers to map the representation, via linear or nonlinear transformations, into the desired output dimensionality. Conversely, for tasks that require prediction at the pixel or voxel level, it is necessary to upsample the intermediate representations back to the original resolution. This is typically also followed by a task-specific decoding mapping that ensures the output is of appropriate size and structure. In cases where the prediction goal is to assign each pixel or voxel a label from a predefined set, the task is formally defined as segmentation.

Regardless of the specific prediction task, MRI data can be processed using either 2D or 3D analysis approaches. In 2D analysis, individual slices or cross-sections of the volume are processed separately [13], with final predictions potentially obtained by aggregating the outputs across slices. This enables direct application of standard 2D CNN or ViT layers. Alternatively, full 3D analysis may be performed, which requires the use of 3D convolutions in CNN architectures and 3D patches in transformer-based attention mechanisms to capture volumetric spatial dependencies.

In the field of medical image segmentation, the most widely adopted architecture is based on the U-Net framework [187,188], which, in the case of 3D data, is sometimes referred to as V-Net [189]. In this architecture, the downsampling (encoder) and upsampling (decoder) paths follow a symmetrical design, in which the spatial resolution of the feature representations is progressively reduced and then restored. Numerous modifications of this architecture can be found in the literature, including the incorporation of residual connections and specialized blocks within the encoder and decoder [13,14,55,65], as well as variations in training strategies and usage paradigms [132,136,150,151]. Other CNN-based architectures such as FCN [190], DeepLab [191], or SegNet [192] are considerably less common in the context of the problems addressed in this work. This is primarily due to their typically lower segmentation accuracy, particularly in delineating fine object boundaries, and their often-higher demand for large, annotated datasets, which are challenging to obtain in medical imaging.

With regard to the use of attention mechanisms in medical image segmentation, hybrid approaches that combine convolutional layers with the U-Net architecture are currently the most prevalent. In these methods, a transformer module is typically integrated as a bottleneck between the encoder and decoder to enable global context modeling, which standard convolutional networks struggle to capture due to their inherently local receptive fields. Notable examples of this approach include TransUNet [193] and TransBTS [194]. Alternatively, in models such as UNETR [195], the transformer is employed directly as the encoder.

When developing algorithms for medical image segmentation, it is essential to define appropriate performance metrics to evaluate their outcomes. In the case of multi-label segmentation, the evaluation problem can be reformulated as a series of binary segmentation tasks, where each label is treated as the foreground and all remaining classes are considered background. By performing such an assessment for each label separately, a final evaluation can be obtained by aggregating the results using a suitable averaging strategy (e.g., macro, micro, or weighted averaging). The choice of averaging method becomes particularly important in the presence of class imbalance.

Since image segmentation is essentially a pixel- or voxel-wise classification task, standard classification metrics are commonly employed in the literature. These include class accuracy, precision, recall (sensitivity), specificity, the F1 score (equivalent to the Dice coefficient), and other metrics derived from the confusion matrix [132,150,151]. Additionally, the Jaccard index (also known as Intersection over Union, IoU) is frequently used, as it provides an intuitive measure of the spatial (surface or volumetric) overlap between the predicted and ground truth segmentations. For deep-learning neural network (DLNN) models, which typically output class probability estimates, metrics such as the area under the curve (AUC) for the receiver operating characteristic (ROC) and precision–recall (PR) curves are also relevant [55,65]. Finally, due to the inherently geometric nature of the segmentation task, some studies incorporate distance-based metrics that quantify the spatial discrepancy between predicted and ground truth boundaries or surfaces. These include the Hausdorff Distance (HD) [196], its more robust variant HD95, and the Average Symmetric Surface Distance (ASSD) [197].

Also, the problem of poor DLNN explainability is nowadays more and more often raised as a crucial issue. There are many reasons why neural networks may fail. The problem may be located in a model itself (poor architecture choice may lead to overfitting/underfitting or to vulnerability to unintentional attacks) or in the training procedure (bad selection of loss function and/or optimizer). There are naturally machine-learning techniques that allow us to avoid most of those issues. Unfortunately, the problem may also be in the data used to prepare models, which can be hard to detect using typical validation procedures. For example, some data distribution shifts may not be foreseen correctly. Moreover, data can contain unexpected biases, which may allow neural networks to learn unexpected correlations. In those cases, models behaving, at first glance, well in the lab can fail in the production environment. In applications where human life and health depend on it, such a situation is unacceptable.

Due to those reasons, techniques belonging to so-called explainable AI (XAI) are currently gaining a lot of interest. The simplest approaches attempt to assign attributions to model inputs. When images are processed, it leads to indicating image areas that were crucial for making a decision by DLNN [198,199]. They allow domain experts, to some extent, to observe what the premises were for the observed outcome. Such methods, however, do not explain the reasoning that led to the obtained result. To some extent, some insight into the decision-making process is given by counterfactual explanations. They try to show the smallest possible and plausible modification in the image, which would lead to a different decision [200,201]. By observing those modifications, physicians can compare them with their medical knowledge and decide if there is any basis for such reasoning. And although both those groups are developing dynamically and cited literature indicates an increasing number of medical applications, it must be emphasized that there is a wide field for further research (especially when it comes to counterfactuals and brain images).

With explanations of DLNNs, and in particular CNNs, there is one additional problem. Since CNNs operate on pixels/voxels, interpretation of their working as well as interpretation of provided explanations may be limited. The reason for that is not only the computational complexity but also the fact that domain experts analyzing images do not operate on such small structural elements. Physicians would rather take into account anatomical structures or at least their fragments (semantically coherent regions). It means that such elements of higher granularity need to be identified first and later used, taking into account their meaning and spatial relationships between them, for reasoning similar to that of humans. Several approaches to that problem can be found in the literature where other than CNN-based DLNNs are used. An example can be, although the motivation for its creation was slightly different, Vision Transformer (ViT), where the image is split into square patches. ViT, using an attention mechanism, learns the influence of every patch in the image on any other patch and uses this information for final decisions [202,203]. This process can be viewed as a message passing mechanism between all the patches, which means that a complete graph is considered for computations, and makes the whole reasoning quite complex. To mitigate that problem, graph neural networks (GNNs), a generalization of CNN-based and attention-based models, can be of use. Such an approach is used, for example, in Vision GNN [204].

It is worth noting that, since GNNs can process any graph structure, they have great potential as a tool for operating on semantic image components, leading to models that better reflect human-like reasoning and are consequently easier to understand and trust.

To sum up, all the presented trends in AI fit perfectly with the General-to-Specific approach postulated in this work. On the one hand, they support involving domain experts in the process of DLNN training, and on the other hand, they provide tools for their careful validation, which in medicine is of special importance.

### Study Limitations

One limitation of this study is the absence of a quantitative synthesis as well as a formal risk of bias assessment, which is standard in systematic reviews but not required for scoping reviews. As our review followed the PRISMA-ScR guidelines, the focus was on mapping the scope and nature of existing evidence rather than evaluating study quality or clinical outcomes. Additionally, the review was not registered in PROSPERO, as current policies exclude scoping reviews from eligibility. We did not define specific clinical outcomes like diagnostic accuracy, as this would not align with the descriptive aims of the study. These methodological choices reflect the exploratory nature of scoping reviews in emerging and diverse fields such as AI in neuro-oncology.

Furthermore, our review included articles published up to April 2024. The remaining time was dedicated to screening a substantial number of studies and thoroughly analyzing the included articles. Due to the nature of this type of research, a certain degree of time lag is inevitable, which means the review may not fully reflect the most recent developments.

Finally, while the reported accuracy of the AI model is encouraging, it has not yet been tested across the full spectrum of real-world variability. In particular, comprehensive multicenter validation is lacking, which is essential to confirm robustness across diverse patient populations, imaging protocols, and healthcare settings. Our comparison across 1.5 T and 3 T scanners is preliminary and highlights the need for broader validation [205]. The creation of such models and their rigorous multicenter validation—with careful consideration of differing ethical and regulatory requirements—represents a substantial challenge for the near future.

## 5. Conclusions

Recent advancements in artificial intelligence methods have begun to enable differentiation between normal and abnormal CNS imaging findings, distinguishing various pathological entities, and in some cases, even precise tumor classification. Increasingly, these techniques allow highly accurate identification of tumor molecular variants directly from radiological features, a process previously requiring histopathological staining followed by time-consuming molecular analyses. Additionally, AI applications extend into the postoperative phase, particularly in planning radiotherapy, thus significantly enhancing clinical decision-making and patient management. To fully realize this potential, future efforts should prioritize the development of anatomically grounded, interpretable, and clinically integrated AI systems, trained not only to classify but also to understand, support, and ultimately improve real-world neurosurgical decision-making.

## Figures and Tables

**Table 1:** Classification of studies included in our scoping review, organized by main clinical focus and illustrated with representative examples.

“Main” Topic	Examples
Differentiation between specific types of lesions	Glioblastoma from solitary metastatic tumors
	Gliomas from lymphomas
	Glioblastoma, solitary metastases, or CNS lymphomas
	Others
Gliomas	Molecular assessment
	Detection and grading
	Survival prediction
	Pseudoprogression vs. progression
	Combined outcomes
	Others
Metastases	Detection and segmentation
	Lung cancer metastases (differentiation/survival)
	Radiotherapy support/monitoring
	Primary site identification
	Others
Others	Sellar region tumors
	Meningiomas
	Others

**Table 2:** Comparison of both General-to-Specific and Specific-to-General approaches regarding advantages, disadvantages, and application in medicine.

Criterion	General-to-Specific	Specific-to-General
Approach Description	Learning starts with general concepts, rules, or structures, followed by specific cases and exceptions.	Learning begins with concrete examples or observations, from which general patterns or rules are derived.
Advantages	- Structured understanding- Easier to organize knowledge logically - Consistent and scalable models	- Fast results for specific tasks - Effective for small, well-defined problems
Disadvantages	- Slower initial progress - May feel abstract without context - Requires strong domain knowledge early on	- Risk of overfitting to atypical cases - Harder to generalize findings - Models may become opaque or less explainable
Application in Medicine	- Teaching AI normal anatomy and physiology first, then pathology - Aligns with medical education structure	- Training AI on real-world diagnostic cases, including rare tumours - Useful for solving niche clinical problems
Transparency/Interpretability	High—reasoning and decision-making can be traced	Low—model may work, but its logic is unclear (black box effect)
Bias/Error Risk	Lower—less susceptible to noise or irrelevant patterns in the data	Higher—may learn shortcuts or superficial features (e.g., “long hair = female”)
Best Use Cases	- Clinical decision-support systems - Educational models - Radiogenomic predictions	- Image classification of rare diseases - Detection of atypical patterns - Specialized diagnostic tools

## References

[1] Gittleman H.R., Ostrom Q.T., Rouse C.D., Dowling J.A., De Blank P.M., Kruchko C.A., Elder J.B., Rosenfeld S.S., Selman W.R., Sloan A.E. (2015). Trends in Central Nervous System Tumor Incidence Relative to Other Common Cancers in Adults, Adolescents, and Children in the United States, 2000 to 2010. Cancer.

[2] Vernooij M.W., Ikram M.A., Tanghe H.L., Vincent A.J.P.E., Hofman A., Krestin G.P., Niessen W.J., Breteler M.M.B., van der Lugt A. (2007). Incidental Findings on Brain MRI in the General Population. N. Engl. J. Med..

[3] Niewykorzystany Potencjał Wysokospecjalistycznej Aparatury Medycznej—Najwyższa Izba Kontroli. https://www.nik.gov.pl/aktualnosci/niewykorzystany-potencjal-wysokospecjalistycznej-aparatury-medycznej.html.

[4] Mismatch Between Radiologist Shortages, Rising Exam Volumes a Growing Concern in Medical Imaging. https://radiologybusiness.com/topics/healthcare-management/healthcare-staffing/mismatch-between-radiologist-shortages-rising-exam-volumes-growing-concern-medical-imaging.

[5] Myszczynska M.A., Ojamies P.N., Lacoste A.M.B., Neil D., Saffari A., Mead R., Hautbergue G.M., Holbrook J.D., Ferraiuolo L. (2020). Applications of Machine Learning to Diagnosis and Treatment of Neurodegenerative Diseases. Nat. Rev. Neurol..

[6] Puzio T., Matera K., Wiśniewski K., Grobelna M., Wanibuchi S., Jaskólski D.J., Bobeff E.J. (2024). Automated Volumetric Evaluation of Intracranial Compartments and Cerebrospinal Fluid Distribution on Emergency Trauma Head CT Scans to Quantify Mass Effect. Front. Neurosci..

[7] Onakpojeruo E.P., Mustapha M.T., Ozsahin D.U., Ozsahin I. (2024). Enhanced MRI-Based Brain Tumour Classification with a Novel Pix2pix Generative Adversarial Network Augmentation Framework. Brain Commun..

[8] Onakpojeruo E.P., Mustapha M.T., Ozsahin D.U., Ozsahin I. (2024). A Comparative Analysis of the Novel Conditional Deep Convolutional Neural Network Model, Using Conditional Deep Convolutional Generative Adversarial Network-Generated Synthetic and Augmented Brain Tumor Datasets for Image Classification. Brain Sci..

[9] Thillaikkarasi R., Saravanan S. (2019). An Enhancement of Deep Learning Algorithm for Brain Tumor Segmentation Using Kernel Based CNN with M-SVM. J. Med. Syst..

[10] Zhang Q., Ruan G., Yang W., Liu Y., Zhao K., Feng Q., Chen W., Wu E.X., Feng Y. (2019). MRI Gibbs-Ringing Artifact Reduction by Means of Machine Learning Using Convolutional Neural Networks. Magn. Reson. Med..

[11] Khan F., Ayoub S., Gulzar Y., Majid M., Reegu F.A., Mir M.S., Soomro A.B., Elwasila O. (2023). MRI-Based Effective Ensemble Frameworks for Predicting Human Brain Tumor. J. Imaging.

[12] Gauriau R., Bizzo B.C., Kitamura F.C., Junior O.L., Ferraciolli S.F., Macruz F.B.C., Sanchez T.A., Garcia M.R.T., Vedolin L.M., Domingues R.C. (2021). A Deep Learning-Based Model for Detecting Abnormalities on Brain MR Images for Triaging: Preliminary Results from a Multisite Experience. Radiol. Artif. Intell..

[13] Wu S., Li H., Quang D., Guan Y. (2020). Three-Plane-Assembled Deep Learning Segmentation of Gliomas. Radiol. Artif. Intell..

[14] Zhou J., Ye J., Liang Y., Zhao J., Wu Y., Luo S., Lai X., Wang J. (2022). ScSE-NL V-Net: A Brain Tumor Automatic Segmentation Method Based on Spatial and Channel “Squeeze-and-Excitation” Network with Non-Local Block. Front. Neurosci..

[15] Rasheed Z., Ma Y.K., Ullah I., Al Shloul T., Tufail A.B., Ghadi Y.Y., Khan M.Z., Mohamed H.G. (2023). Automated Classification of Brain Tumors from Magnetic Resonance Imaging Using Deep Learning. Brain Sci..

[16] Shen S., Li C., Fan Y., Lu S., Yan Z., Liu H., Zhou H., Zhang Z. (2024). Development and Validation of a Multi-Modality Fusion Deep Learning Model for Differentiating Glioblastoma from Solitary Brain Metastases. Zhong Nan Da Xue Xue Bao Yi Xue Ban.

[17] Shin I., Kim H., Ahn S.S., Sohn B., Bae S., Park J.E., Kim H.S., Lee S.K. (2021). Development and Validation of a Deep Learning-Based Model to Distinguish Glioblastoma from Solitary Brain Metastasis Using Conventional MR Images. AJNR Am. J. Neuroradiol..

[18] Bae S., An C., Ahn S.S., Kim H., Han K., Kim S.W., Park J.E., Kim H.S., Lee S.K. (2020). Robust Performance of Deep Learning for Distinguishing Glioblastoma from Single Brain Metastasis Using Radiomic Features: Model Development and Validation. Sci. Rep..

[19] Zhang Y., Zhang H., Zhang H., Ouyang Y., Su R., Yang W., Huang B. (2023). Glioblastoma and Solitary Brain Metastasis: Differentiation by Integrating Demographic-MRI and Deep-Learning Radiomics Signatures. J. Magn. Reson. Imaging.

[20] Yan Q., Li F., Cui Y., Wang Y., Wang X., Jia W., Liu X., Li Y., Chang H., Shi F. (2023). Discrimination Between Glioblastoma and Solitary Brain Metastasis Using Conventional MRI and Diffusion-Weighted Imaging Based on a Deep Learning Algorithm. J. Digit. Imaging.

[21] Bathla G., Dhruba D.D., Liu Y., Le N.H., Soni N., Zhang H., Mohan S., Roberts-Wolfe D., Rathore S., Sonka M. (2024). Differentiation Between Glioblastoma and Metastatic Disease on Conventional MRI Imaging Using 3D-Convolutional Neural Networks: Model Development and Validation. Acad. Radiol..

[22] Stadlbauer A., Heinz G., Marhold F., Meyer-Bäse A., Ganslandt O., Buchfelder M., Oberndorfer S. (2022). Differentiation of Glioblastoma and Brain Metastases by MRI-Based Oxygen Metabolomic Radiomics and Deep Learning. Metabolites.

[23] Bai J., He M., Gao E., Yang G., Zhang C., Yang H., Dong J., Ma X., Gao Y., Zhang H. (2024). High-Performance Presurgical Differentiation of Glioblastoma and Metastasis by Means of Multiparametric Neurite Orientation Dispersion and Density Imaging (NODDI) Radiomics. Eur. Radiol..

[24] Yun J., Park J.E., Lee H., Ham S., Kim N., Kim H.S. (2019). Radiomic Features and Multilayer Perceptron Network Classifier: A Robust MRI Classification Strategy for Distinguishing Glioblastoma from Primary Central Nervous System Lymphoma. Sci. Rep..

[25] Banzato T., Bernardini M., Cherubini G.B., Zotti A. (2018). A Methodological Approach for Deep Learning to Distinguish between Meningiomas and Gliomas on Canine MR-Images. BMC Vet. Res..

[26] Bhattacharjee S., Prakash D., Kim C.H., Kim H.C., Choi H.K. (2022). Texture, Morphology, and Statistical Analysis to Differentiate Primary Brain Tumors on Two-Dimensional Magnetic Resonance Imaging Scans Using Artificial Intelligence Techniques. Healthc. Inform. Res..

[27] Tariciotti L., Caccavella V.M., Fiore G., Schisano L., Carrabba G., Borsa S., Giordano M., Palmisciano P., Remoli G., Remore L.G. (2022). A Deep Learning Model for Preoperative Differentiation of Glioblastoma, Brain Metastasis and Primary Central Nervous System Lymphoma: A Pilot Study. Front. Oncol..

[28] Liu X., Liu J. (2024). Aided Diagnosis Model Based on Deep Learning for Glioblastoma, Solitary Brain Metastases, and Primary Central Nervous System Lymphoma with Multi-Modal MRI. Biology.

[29] Mahum R., Sharaf M., Hassan H., Liang L., Huang B. (2023). A Robust Brain Tumor Detector Using BiLSTM and Mayfly Optimization and Multi-Level Thresholding. Biomedicines.

[30] Sheng Y., Zhao B., Cheng H., Yu Y., Wang W., Yang Y., Ding Y., Qiu L., Qin Z., Yao Z. (2024). A Convolutional Neural Network Model for Distinguishing Hemangioblastomas from Other Cerebellar-and-Brainstem Tumors Using Contrast-Enhanced MRI. J. Magn. Reson. Imaging.

[31] Pattanaik B.B., Anitha K., Rathore S., Biswas P., Sethy P.K., Behera S.K. (2022). Brain Tumor Magnetic Resonance Images Classification Based Machine Learning Paradigms. Contemp. Oncol..

[32] Asif S., Zhao M., Chen X., Zhu Y. (2023). BMRI-NET: A Deep Stacked Ensemble Model for Multi-Class Brain Tumor Classification from MRI Images. Interdiscip. Sci..

[33] Ali M.U., Hussain S.J., Zafar A., Bhutta M.R., Lee S.W. (2023). WBM-DLNets: Wrapper-Based Metaheuristic Deep Learning Networks Feature Optimization for Enhancing Brain Tumor Detection. Bioengineering.

[34] Abd El-Wahab B.S., Nasr M.E., Khamis S., Ashour A.S. (2023). BTC-FCNN: Fast Convolution Neural Network for Multi-Class Brain Tumor Classification. Health Inf. Sci. Syst..

[35] Rasool M., Ismail N., Boulila W., Ammar A., Samma H., Yafooz W.S., Emara A.H. (2022). A Hybrid Deep Learning Model for Brain Tumour Classification. Entropy.

[36] Ullah N., Javed A., Alhazmi A., Hasnain S.M., Tahir A., Ashraf R. (2023). TumorDetNet: A Unified Deep Learning Model for Brain Tumor Detection and Classification. PLoS ONE.

[37] Mohammed B.A., Al-Ani M.S. (2020). An Efficient Approach to Diagnose Brain Tumors through Deep CNN. Math. Biosci. Eng..

[38] Peng J., Kim D.D., Patel J.B., Zeng X., Huang J., Chang K., Xun X., Zhang C., Sollee J., Wu J. (2022). Deep Learning-Based Automatic Tumor Burden Assessment of Pediatric High-Grade Gliomas, Medulloblastomas, and Other Leptomeningeal Seeding Tumors. Neuro. Oncol..

[39] Chakrabarty S., Sotiras A., Milchenko M., Lamontagne P., Hileman M., Marcus D. (2021). MRI-Based Identification and Classification of Major Intracranial Tumor Types by Using a 3D Convolutional Neural Network: A Retrospective Multi-Institutional Analysis. Radiol. Artif. Intell..

[40] Stadlbauer A., Marhold F., Oberndorfer S., Heinz G., Buchfelder M., Kinfe T.M., Meyer-Bäse A. (2022). Radiophysiomics: Brain Tumors Classification by Machine Learning and Physiological MRI Data. Cancers.

[41] Windisch P., Weber P., Fürweger C., Ehret F., Kufeld M., Zwahlen D., Muacevic A. (2020). Implementation of Model Explainability for a Basic Brain Tumor Detection Using Convolutional Neural Networks on MRI Slices. Neuroradiology.

[42] Ye N., Yang Q., Chen Z., Teng C., Liu P., Liu X., Xiong Y., Lin X., Li S., Li X. (2022). Classification of Gliomas and Germinomas of the Basal Ganglia by Transfer Learning. Front. Oncol..

[43] Pan Y., Huang W., Lin Z., Zhu W., Zhou J., Wong J., Ding Z. Brain Tumor Grading Based on Neural Networks and Convolutional Neural Networks. Proceedings of the 2015 37th Annual International Conference of the IEEE Engineering in Medicine and Biology Society (EMBC).

[44] Tang W., Zhang H., Yu P., Kang H., Zhang R. MMMNA-Net for Overall Survival Time Prediction of Brain Tumor Patients. Proceedings of the 2022 44th Annual International Conference of the IEEE Engineering in Medicine & Biology Society (EMBC).

[45] Sun L., Zhang S., Chen H., Luo L. (2019). Brain Tumor Segmentation and Survival Prediction Using Multimodal MRI Scans with Deep Learning. Front. Neurosci..

[46] Nie D., Lu J., Zhang H., Adeli E., Wang J., Yu Z., Liu L.Y., Wang Q., Wu J., Shen D. (2019). Multi-Channel 3D Deep Feature Learning for Survival Time Prediction of Brain Tumor Patients Using Multi-Modal Neuroimages. Sci. Rep..

[47] Nie D., Zhang H., Adeli E., Liu L., Shen D. (2016). 3D Deep Learning for Multi-Modal Imaging-Guided Survival Time Prediction of Brain Tumor Patients. Med. Image Comput. Comput. Assist. Interv..

[48] Quon J.L., Chen L.C., Kim L., Grant G.A., Edwards M.S.B., Cheshier S.H., Yeom K.W. (2020). Deep Learning for Automated Delineation of Pediatric Cerebral Arteries on Pre-Operative Brain Magnetic Resonance Imaging. Front. Surg..

[49] Zhang F., Hoffmann N., Karayumak S.C., Rathi Y., Golby A.J., O’Donnell L.J. (2019). Deep White Matter Analysis: Fast, Consistent Tractography Segmentation across Populations and DMRI Acquisitions. Med. Image Comput. Comput. Assist. Interv..

[50] Young F., Aquilina K., Seunarine K.K., Mancini L., Clark C.A., Clayden J.D. (2024). Fibre Orientation Atlas Guided Rapid Segmentation of White Matter Tracts. Hum. Brain Mapp..

[51] Meesters S., Landers M., Rutten G.J., Florack L. (2023). Subject-Specific Automatic Reconstruction of White Matter Tracts. J. Digit. Imaging.

[52] Lucena O., Lavrador J.P., Irzan H., Semedo C., Borges P., Vergani F., Granados A., Sparks R., Ashkan K., Ourselin S. (2023). Assessing Informative Tract Segmentation and NTMS for Pre-Operative Planning. J. Neurosci. Methods.

[53] Luckett P.H., Park K.Y., Lee J.J., Lenze E.J., Wetherell J.L., Eyler L.T., Snyder A.Z., Ances B.M., Shimony J.S., Leuthardt E.C. (2023). Data-Efficient Resting-State Functional Magnetic Resonance Imaging Brain Mapping with Deep Learning. J. Neurosurg..

[54] Ge C., Gu I.Y.H., Jakola A.S., Yang J. (2020). Deep Semi-Supervised Learning for Brain Tumor Classification. BMC Med. Imaging.

[55] Kihira S., Mei X., Mahmoudi K., Liu Z., Dogra S., Belani P., Tsankova N., Hormigo A., Fayad Z.A., Doshi A. (2022). U-Net Based Segmentation and Characterization of Gliomas. Cancers.

[56] Cao M., Suo S., Zhang X., Wang X., Xu J., Yang W., Zhou Y. (2021). Qualitative and Quantitative MRI Analysis in IDH1 Genotype Prediction of Lower-Grade Gliomas: A Machine Learning Approach. Biomed Res. Int..

[57] Choi Y.S., Bae S., Chang J.H., Kang S.G., Kim S.H., Kim J., Rim T.H., Choi S.H., Jain R., Lee S.K. (2021). Fully Automated Hybrid Approach to Predict the IDH Mutation Status of Gliomas via Deep Learning and Radiomics. Neuro. Oncol..

[58] Kawaguchi R.K., Takahashi M., Miyake M., Kinoshita M., Takahashi S., Ichimura K., Hamamoto R., Narita Y., Sese J. (2021). Assessing Versatile Machine Learning Models for Glioma Radiogenomic Studies across Hospitals. Cancers.

[59] Zhao Y., Wang W., Ji Y., Guo Y., Duan J., Liu X., Yan D., Liang D., Li W., Zhang Z. (2024). Computational Pathology for Prediction of Isocitrate Dehydrogenase Gene Mutation from Whole Slide Images in Adult Patients with Diffuse Glioma. Am. J. Pathol..

[60] Bangalore Yogananda C.G., Wagner B.C., Truong N.C.D., Holcomb J.M., Reddy D.D., Saadat N., Hatanpaa K.J., Patel T.R., Fei B., Lee M.D. (2023). MRI-Based Deep Learning Method for Classification of IDH Mutation Status. Bioengineering.

[61] Safari M., Beiki M., Ameri A., Toudeshki S.H., Fatemi A., Archambault L. (2022). Shuffle-ResNet: Deep Learning for Predicting LGG IDH1 Mutation from Multicenter Anatomical MRI Sequences. Biomed. Phys. Eng. Express.

[62] Shi X., Zhang X., Iwamoto Y., Cheng J., Bai J., Zhao G., Chen Y.W. An Intra- and Inter-Modality Fusion Model Using MR Images for Prediction of Glioma Isocitrate Dehydrogenase (IDH) Mutation. Proceedings of the 2022 44th Annual International Conference of the IEEE Engineering in Medicine & Biology Society (EMBC).

[63] Zeng H., Xing Z., Gao F., Wu Z., Huang W., Su Y., Chen Z., Cai S., Cao D., Cai C. (2022). A Multimodal Domain Adaptive Segmentation Framework for IDH Genotype Prediction. Int. J. Comput. Assist. Radiol. Surg..

[64] Park J.E., Eun D., Kim H.S., Lee D.H., Jang R.W., Kim N. (2021). Generative Adversarial Network for Glioblastoma Ensures Morphologic Variations and Improves Diagnostic Model for Isocitrate Dehydrogenase Mutant Type. Sci. Rep..

[65] Choi Y., Nam Y., Lee Y.S., Kim J., Ahn K.J., Jang J., Shin N.Y., Kim B.S., Jeon S.S. (2020). IDH1 Mutation Prediction Using MR-Based Radiomics in Glioblastoma: Comparison between Manual and Fully Automated Deep Learning-Based Approach of Tumor Segmentation. Eur. J. Radiol..

[66] Wei Y., Chen X., Zhu L., Zhang L., Schonlieb C.B., Price S., Li C. (2023). Multi-Modal Learning for Predicting the Genotype of Glioma. IEEE Trans. Med. Imaging.

[67] Nalawade S., Murugesan G.K., Vejdani-Jahromi M., Fisicaro R.A., Bangalore Yogananda C.G., Wagner B., Mickey B., Maher E., Pinho M.C., Fei B. (2019). Classification of Brain Tumor Isocitrate Dehydrogenase Status Using MRI and Deep Learning. J. Med. Imaging.

[68] Choi K.S., Choi S.H., Jeong B. (2019). Prediction of IDH Genotype in Gliomas with Dynamic Susceptibility Contrast Perfusion MR Imaging Using an Explainable Recurrent Neural Network. Neuro. Oncol..

[69] Chang K., Bai H.X., Zhou H., Su C., Bi W.L., Agbodza E., Kavouridis V.K., Senders J.T., Boaro A., Beers A. (2018). Residual Convolutional Neural Network for the Determination of IDH Status in Low- and High-Grade Gliomas from MR Imaging. Clin. Cancer Res..

[70] Yan J., Zhang S., Sun Q., Wang W., Duan W., Wang L., Ding T., Pei D., Sun C., Wang W. (2022). Predicting 1p/19q Co-Deletion Status from Magnetic Resonance Imaging Using Deep Learning in Adult-Type Diffuse Lower-Grade Gliomas: A Discovery and Validation Study. Lab. Investig..

[71] Yogananda C.G.B., Shah B.R., Yu F.F., Pinho M.C., Nalawade S.S., Murugesan G.K., Wagner B.C., Mickey B., Patel T.R., Fei B. (2020). A Novel Fully Automated MRI-Based Deep-Learning Method for Classification of 1p/19q Co-Deletion Status in Brain Gliomas. Neuro-Oncol. Adv..

[72] Ge C., Gu I.Y.H., Jakola A.S., Yang J. Deep Learning and Multi-Sensor Fusion for Glioma Classification Using Multistream 2D Convolutional Networks. Proceedings of the 2018 40th Annual International Conference of the IEEE Engineering in Medicine and Biology Society (EMBC).

[73] Capuozzo S., Gravina M., Gatta G., Marrone S., Sansone C. (2022). A Multimodal Knowledge-Based Deep Learning Approach for MGMT Promoter Methylation Identification. J. Imaging.

[74] Chen S., Xu Y., Ye M., Li Y., Sun Y., Liang J., Lu J., Wang Z., Zhu Z., Zhang X. (2022). Predicting MGMT Promoter Methylation in Diffuse Gliomas Using Deep Learning with Radiomics. J. Clin. Med..

[75] Kim B.H., Lee H., Choi K.S., Nam J.G., Park C.K., Park S.H., Chung J.W., Choi S.H. (2022). Validation of MRI-Based Models to Predict MGMT Promoter Methylation in Gliomas: BraTS 2021 Radiogenomics Challenge. Cancers.

[76] Usuzaki T., Takahashi K., Inamori R., Morishita Y., Shizukuishi T., Takagi H., Ishikuro M., Obara T., Takase K. (2024). Identifying Key Factors for Predicting O6-Methylguanine-DNA Methyltransferase Status in Adult Patients with Diffuse Glioma: A Multimodal Analysis of Demographics, Radiomics, and MRI by Variable Vision Transformer. Neuroradiology.

[77] Shi X., Li Y., Cheng J., Bai J., Zhao G., Chen Y.W. Multi-Task Model for Glioma Segmentation and Isocitrate Dehydrogenase Status Prediction Using Global and Local Features. Proceedings of the 2023 45th Annual International Conference of the IEEE Engineering in Medicine & Biology Society (EMBC).

[78] Saeed N., Ridzuan M., Alasmawi H., Sobirov I., Yaqub M. (2023). MGMT Promoter Methylation Status Prediction Using MRI Scans? An Extensive Experimental Evaluation of Deep Learning Models. Med. Image Anal..

[79] Robinet L., Siegfried A., Roques M., Berjaoui A., Cohen-Jonathan Moyal E. (2023). MRI-Based Deep Learning Tools for MGMT Promoter Methylation Detection: A Thorough Evaluation. Cancers.

[80] Qureshi S.A., Hussain L., Ibrar U., Alabdulkreem E., Nour M.K., Alqahtani M.S., Nafie F.M., Mohamed A., Mohammed G.P., Duong T.Q. (2023). Radiogenomic Classification for MGMT Promoter Methylation Status Using Multi-Omics Fused Feature Space for Least Invasive Diagnosis through MpMRI Scans. Sci. Rep..

[81] Saxena S., Jena B., Mohapatra B., Gupta N., Kalra M., Scartozzi M., Saba L., Suri J.S. (2023). Fused Deep Learning Paradigm for the Prediction of O6-Methylguanine-DNA Methyltransferase Genotype in Glioblastoma Patients: A Neuro-Oncological Investigation. Comput. Biol. Med..

[82] Faghani S., Khosravi B., Moassefi M., Conte G.M., Erickson B.J. (2023). A Comparison of Three Different Deep Learning-Based Models to Predict the MGMT Promoter Methylation Status in Glioblastoma Using Brain MRI. J. Digit. Imaging.

[83] Chen X., Zeng M., Tong Y., Zhang T., Fu Y., Li H., Zhang Z., Cheng Z., Xu X., Yang R. (2020). Automatic Prediction of MGMT Status in Glioblastoma via Deep Learning-Based MR Image Analysis. Biomed Res. Int..

[84] Crisi G., Filice S. (2020). Predicting MGMT Promoter Methylation of Glioblastoma from Dynamic Susceptibility Contrast Perfusion: A Radiomic Approach. J. Neuroimaging.

[85] Yogananda C.G.B., Shah B.R., Nalawade S.S., Murugesan G.K., Yu F.F., Pinho M.C., Wagner B.C., Mickey B., Patel T.R., Fei B. (2021). MRI-Based Deep-Learning Method for Determining Glioma MGMT Promoter Methylation Status. AJNR Am. J. Neuroradiol..

[86] Han L., Kamdar M.R. (2018). MRI to MGMT: Predicting Methylation Status in Glioblastoma Patients Using Convolutional Recurrent Neural Networks. Pacific Symp. Biocomput..

[87] Korfiatis P., Kline T.L., Lachance D.H., Parney I.F., Buckner J.C., Erickson B.J. (2017). Residual Deep Convolutional Neural Network Predicts MGMT Methylation Status. J. Digit. Imaging.

[88] Tillmanns N., Lost J., Tabor J., Vasandani S., Vetsa S., Marianayagam N., Yalcin K., Erson-Omay E.Z., von Reppert M., Jekel L. (2023). Application of Novel PACS-Based Informatics Platform to Identify Imaging Based Predictors of CDKN2A Allelic Status in Glioblastomas. Sci. Rep..

[89] Li J., Zhang P., Qu L., Sun T., Duan Y., Wu M., Weng J., Li Z., Gong X., Liu X. (2023). Deep Learning for Noninvasive Assessment of H3 K27M Mutation Status in Diffuse Midline Gliomas Using MR Imaging. J. Magn. Reson. Imaging.

[90] Huang B., Zhang Y., Mao Q., Ju Y., Liu Y., Su Z., Lei Y., Ren Y. (2023). Deep Learning-Based Prediction of H3K27M Alteration in Diffuse Midline Gliomas Based on Whole-Brain MRI. Cancer Med..

[91] Chakrabarty S., Lamontagne P., Shimony J., Marcus D.S., Sotiras A. (2023). MRI-Based Classification of IDH Mutation and 1p/19q Codeletion Status of Gliomas Using a 2.5D Hybrid Multi-Task Convolutional Neural Network. Neuro-Oncol. Adv..

[92] Decuyper M., Bonte S., Deblaere K., Van Holen R. (2021). Automated MRI Based Pipeline for Segmentation and Prediction of Grade, IDH Mutation and 1p19q Co-Deletion in Glioma. Comput. Med. Imaging Graph..

[93] Kihira S., Derakhshani A., Leung M., Mahmoudi K., Bauer A., Zhang H., Polson J., Arnold C., Tsankova N.M., Hormigo A. (2023). Multi-Parametric Radiomic Model to Predict 1p/19q Co-Deletion in Patients with IDH-1 Mutant Glioma: Added Value to the T2-FLAIR Mismatch Sign. Cancers.

[94] Nalawade S.S., Yu F.F., Bangalore Yogananda C.G., Murugesan G.K., Shah B.R., Pinho M.C., Wagner B.C., Xi Y., Mickey B., Patel T.R. (2022). Brain Tumor IDH, 1p/19q, and MGMT Molecular Classification Using MRI-Based Deep Learning: An Initial Study on the Effect of Motion and Motion Correction. J. Med. Imaging.

[95] Buz-Yalug B., Turhan G., Cetin A.I., Dindar S.S., Danyeli A.E., Yakicier C., Pamir M.N., Özduman K., Dincer A., Ozturk-Isik E. (2024). Identification of IDH and TERTp Mutations Using Dynamic Susceptibility Contrast MRI with Deep Learning in 162 Gliomas. Eur. J. Radiol..

[96] Zhang L., Wang R., Gao J., Tang Y., Xu X., Kan Y., Cao X., Wen Z., Liu Z., Cui S. (2024). A Novel MRI-Based Deep Learning Networks Combined with Attention Mechanism for Predicting CDKN2A/B Homozygous Deletion Status in IDH-Mutant Astrocytoma. Eur. Radiol..

[97] Calabrese E., Villanueva-Meyer J.E., Cha S. (2020). A Fully Automated Artificial Intelligence Method for Non-Invasive, Imaging-Based Identification of Genetic Alterations in Glioblastomas. Sci. Rep..

[98] Rui W., Zhang S., Shi H., Sheng Y., Zhu F., Yao Y., Chen X., Cheng H., Zhang Y., Aili A. (2023). Deep Learning-Assisted Quantitative Susceptibility Mapping as a Tool for Grading and Molecular Subtyping of Gliomas. Phenomics.

[99] Ali M.B., Gu I.Y.H., Berger M.S., Pallud J., Southwell D., Widhalm G., Roux A., Vecchio T.G., Jakola A.S. (2020). Domain Mapping and Deep Learning from Multiple MRI Clinical Datasets for Prediction of Molecular Subtypes in Low Grade Gliomas. Brain Sci..

[100] Li Y., Wei D., Liu X., Fan X., Wang K., Li S., Zhang Z., Ma K., Qian T., Jiang T. (2022). Molecular Subtyping of Diffuse Gliomas Using Magnetic Resonance Imaging: Comparison and Correlation between Radiomics and Deep Learning. Eur. Radiol..

[101] Xu Q., Xu Q.Q., Shi N., Dong L.N., Zhu H., Xu K. (2022). A Multitask Classification Framework Based on Vision Transformer for Predicting Molecular Expressions of Glioma. Eur. J. Radiol..

[102] Tak D., Ye Z., Zapaischykova A., Zha Y., Boyd A., Vajapeyam S., Chopra R., Hayat H., Prabhu S.P., Liu K.X. (2024). Noninvasive Molecular Subtyping of Pediatric Low-Grade Glioma with Self-Supervised Transfer Learning. Radiol. Artif. Intell..

[103] Matsui Y., Maruyama T., Nitta M., Saito T., Tsuzuki S., Tamura M., Kusuda K., Fukuya Y., Asano H., Kawamata T. (2020). Prediction of Lower-Grade Glioma Molecular Subtypes Using Deep Learning. J. Neurooncol..

[104] Liu L., Chang J., Zhang P., Qiao H., Xiong S. (2023). SASG-GCN: Self-Attention Similarity Guided Graph Convolutional Network for Multi-Type Lower-Grade Glioma Classification. IEEE J. Biomed. Health Inform..

[105] Buda M., Saha A., Mazurowski M.A. (2019). Association of Genomic Subtypes of Lower-Grade Gliomas with Shape Features Automatically Extracted by a Deep Learning Algorithm. Comput. Biol. Med..

[106] Yoon J., Baek N., Yoo R.E., Choi S.H., Kim T.M., Park C.K., Park S.H., Won J.K., Lee J.H., Lee S.T. (2024). Added Value of Dynamic Contrast-Enhanced MR Imaging in Deep Learning-Based Prediction of Local Recurrence in Grade 4 Adult-Type Diffuse Gliomas Patients. Sci. Rep..

[107] Guo P., Unberath M., Heo H.Y., Eberhart C.G., Lim M., Blakeley J.O., Jiang S. (2022). Learning-Based Analysis of Amide Proton Transfer-Weighted MRI to Identify True Progression in Glioma Patients. NeuroImage Clin..

[108] Peeken J.C., Molina-Romero M., Diehl C., Menze B.H., Straube C., Meyer B., Zimmer C., Wiestler B., Combs S.E. (2019). Deep Learning Derived Tumor Infiltration Maps for Personalized Target Definition in Glioblastoma Radiotherapy. Radiother. Oncol..

[109] Ermiş E., Jungo A., Poel R., Blatti-Moreno M., Meier R., Knecht U., Aebersold D.M., Fix M.K., Manser P., Reyes M. (2020). Fully Automated Brain Resection Cavity Delineation for Radiation Target Volume Definition in Glioblastoma Patients Using Deep Learning. Radiat. Oncol..

[110] Shim K.Y., Chung S.W., Jeong J.H., Hwang I., Park C.K., Kim T.M., Park S.H., Won J.K., Lee J.H., Lee S.T. (2021). Radiomics-Based Neural Network Predicts Recurrence Patterns in Glioblastoma Using Dynamic Susceptibility Contrast-Enhanced MRI. Sci. Rep..

[111] Lee J., Wang N., Turk S., Mohammed S., Lobo R., Kim J., Liao E., Camelo-Piragua S., Kim M., Junck L. (2020). Discriminating Pseudoprogression and True Progression in Diffuse Infiltrating Glioma Using Multi-Parametric MRI Data through Deep Learning. Sci. Rep..

[112] Akbari H., Rathore S., Bakas S., Nasrallah M.P., Shukla G., Mamourian E., Rozycki M., Bagley S.J., Rudie J.D., Flanders A.E. (2020). Histopathology-Validated Machine Learning Radiographic Biomarker for Noninvasive Discrimination between True Progression and Pseudo-Progression in Glioblastoma. Cancer.

[113] Bacchi S., Zerner T., Dongas J., Asahina A.T., Abou-Hamden A., Otto S., Oakden-Rayner L., Patel S. (2019). Deep Learning in the Detection of High-Grade Glioma Recurrence Using Multiple MRI Sequences: A Pilot Study. J. Clin. Neurosci..

[114] Moassefi M., Faghani S., Conte G.M., Kowalchuk R.O., Vahdati S., Crompton D.J., Perez-Vega C., Cabreja R.A.D., Vora S.A., Quiñones-Hinojosa A. (2022). A Deep Learning Model for Discriminating True Progression from Pseudoprogression in Glioblastoma Patients. J. Neurooncol..

[115] Zhu J., Ye J., Dong L., Ma X., Tang N., Xu P., Jin W., Li R., Yang G., Lai X. (2023). Non-Invasive Prediction of Overall Survival Time for Glioblastoma Multiforme Patients Based on Multimodal MRI Radiomics. Int. J. Imaging Syst. Technol..

[116] Luckett P.H., Olufawo M., Lamichhane B., Park K.Y., Dierker D., Verastegui G.T., Yang P., Kim A.H., Chheda M.G., Snyder A.Z. (2023). Predicting Survival in Glioblastoma with Multimodal Neuroimaging and Machine Learning. J. Neurooncol..

[117] Yun J., Yun S., Park J.E., Cheong E.N., Park S.Y., Kim N., Kim H.S. (2023). Deep Learning of Time-Signal Intensity Curves from Dynamic Susceptibility Contrast Imaging Enables Tissue Labeling and Prediction of Survival in Glioblastoma. AJNR Am. J. Neuroradiol..

[118] Shaheen A., Bukhari S.T., Nadeem M., Burigat S., Bagci U., Mohy-ud-Din H. (2022). Overall Survival Prediction of Glioma Patients With Multiregional Radiomics. Front. Neurosci..

[119] Tang Z., Cao H., Xu Y., Yang Q., Wang J., Zhang H. (2022). Overall Survival Time Prediction for Glioblastoma Using Multimodal Deep KNN. Phys. Med. Biol..

[120] Moya-Sáez E., Navarro-González R., Cepeda S., Pérez-Núñez Á., de Luis-García R., Aja-Fernández S., Alberola-López C. (2022). Synthetic MRI Improves Radiomics-Based Glioblastoma Survival Prediction. NMR Biomed..

[121] Li Z.C., Yan J., Zhang S., Liang C., Lv X., Zou Y., Zhang H., Liang D., Zhang Z., Chen Y. (2022). Glioma Survival Prediction from Whole-Brain MRI without Tumor Segmentation Using Deep Attention Network: A Multicenter Study. Eur. Radiol..

[122] Ben Ahmed K., Hall L.O., Goldgof D.B., Gatenby R. (2022). Ensembles of Convolutional Neural Networks for Survival Time Estimation of High-Grade Glioma Patients from Multimodal MRI. Diagnostics.

[123] Fu X., Chen C., Li D. (2021). Survival Prediction of Patients Suffering from Glioblastoma Based on Two-Branch DenseNet Using Multi-Channel Features. Int. J. Comput. Assist. Radiol. Surg..

[124] Liu J., Cong C., Zhang J., Qiao J., Guo H., Wu H., Sang Z., Kang H., Fang J., Zhang W. (2024). Multimodel Habitats Constructed by Perfusion and/or Diffusion MRI Predict Isocitrate Dehydrogenase Mutation Status and Prognosis in High-Grade Gliomas. Clin. Radiol..

[125] Li X., Strasser B., Neuberger U., Vollmuth P., Bendszus M., Wick W., Dietrich J., Batchelor T.T., Cahill D.P., Andronesi O.C. (2022). Deep Learning Super-Resolution Magnetic Resonance Spectroscopic Imaging of Brain Metabolism and Mutant Isocitrate Dehydrogenase Glioma. Neuro-Oncol. Adv..

[126] Kamble A.N., Agrawal N.K., Koundal S., Bhargava S., Kamble A.N., Joyner D.A., Kalelioglu T., Patel S.H., Jain R. (2023). Imaging-Based Stratification of Adult Gliomas Prognosticates Survival and Correlates with the 2021 WHO Classification. Neuroradiology.

[127] Ali M.B., Gu I.Y.-H., Lidemar A., Berger M.S., Widhalm G., Jakola A.S. (2022). Prediction of Glioma-Subtypes: Comparison of Performance on a DL Classifier Using Bounding Box Areas versus Annotated Tumors. BMC Biomed. Eng..

[128] Van Der Voort S.R., Incekara F., Wijnenga M.M.J., Kapsas G., Gahrmann R., Schouten J.W., Nandoe Tewarie R., Lycklama G.J., De Witt Hamer P.C., Eijgelaar R.S. (2023). Combined Molecular Subtyping, Grading, and Segmentation of Glioma Using Multi-Task Deep Learning. Neuro. Oncol..

[129] Hsu D.G., Ballangrud Å., Shamseddine A., Deasy J.O., Veeraraghavan H., Cervino L., Beal K., Aristophanous M. (2021). Automatic Segmentation of Brain Metastases Using T1 Magnetic Resonance and Computed Tomography Images. Phys. Med. Biol..

[130] Chartrand G., Emiliani R.D., Pawlowski S.A., Markel D.A., Bahig H., Cengarle-Samak A., Rajakesari S., Lavoie J., Ducharme S., Roberge D. (2022). Automated Detection of Brain Metastases on T1-Weighted MRI Using a Convolutional Neural Network: Impact of Volume Aware Loss and Sampling Strategy. J. Magn. Reson. Imaging.

[131] Amemiya S., Takao H., Kato S., Yamashita H., Sakamoto N., Abe O. (2022). Feature-Fusion Improves MRI Single-Shot Deep Learning Detection of Small Brain Metastases. J. Neuroimaging.

[132] Park Y.W., Jun Y., Lee Y., Han K., An C., Ahn S.S., Hwang D., Lee S.K. (2021). Robust Performance of Deep Learning for Automatic Detection and Segmentation of Brain Metastases Using Three-Dimensional Black-Blood and Three-Dimensional Gradient Echo Imaging. Eur. Radiol..

[133] Dikici E., Ryu J.L., Demirer M., Bigelow M., White R.D., Slone W., Erdal B.S., Prevedello L.M. (2020). Automated Brain Metastases Detection Framework for T1-Weighted Contrast-Enhanced 3D MRI. IEEE J. Biomed. Health Inform..

[134] Jiao T., Li F., Cui Y., Wang X., Li B., Shi F., Xia Y., Zhou Q., Zeng Q. (2023). Deep Learning with an Attention Mechanism for Differentiating the Origin of Brain Metastasis Using MR Images. J. Magn. Reson. Imaging.

[135] Lyu Q., Namjoshi S.V., McTyre E., Topaloglu U., Barcus R., Chan M.D., Cramer C.K., Debinski W., Gurcan M.N., Lesser G.J. (2022). A Transformer-Based Deep-Learning Approach for Classifying Brain Metastases into Primary Organ Sites Using Clinical Whole-Brain MRI Images. Patterns.

[136] Jalalifar A., Soliman H., Sahgal A., Sadeghi-Naini A. A Cascaded Deep-Learning Framework for Segmentation of Metastatic Brain Tumors Before and After Stereotactic Radiation Therapy. Proceedings of the 2020 42nd Annual International Conference of the IEEE Engineering in Medicine & Biology Society (EMBC).

[137] Wang J.Y., Qu V., Hui C., Sandhu N., Mendoza M.G., Panjwani N., Chang Y.C., Liang C.H., Lu J.T., Wang L. (2023). Stratified Assessment of an FDA-Cleared Deep Learning Algorithm for Automated Detection and Contouring of Metastatic Brain Tumors in Stereotactic Radiosurgery. Radiat. Oncol..

[138] Liu Y., Stojadinovic S., Hrycushko B., Wardak Z., Lau S., Lu W., Yan Y., Jiang S.B., Zhen X., Timmerman R. (2017). A Deep Convolutional Neural Network-Based Automatic Delineation Strategy for Multiple Brain Metastases Stereotactic Radiosurgery. PLoS ONE.

[139] Hsu D.G., Ballangrud Å., Prezelski K., Swinburne N.C., Young R., Beal K., Deasy J.O., Cerviño L., Aristophanous M. (2023). Automatically Tracking Brain Metastases after Stereotactic Radiosurgery. Phys. Imaging Radiat. Oncol..

[140] Xue J., Wang B., Ming Y., Liu X., Jiang Z., Wang C., Liu X., Chen L., Qu J., Xu S. (2020). Deep Learning-Based Detection and Segmentation-Assisted Management of Brain Metastases. Neuro. Oncol..

[141] Wang T.W., Chao H.S., Chiu H.Y., Lu C.F., Liao C.Y., Lee Y., Chen J.R., Shiao T.H., Chen Y.M., Wu Y. (2024). Te Radiomics of Metastatic Brain Tumor as a Predictive Image Biomarker of Progression-Free Survival in Patients with Non-Small-Cell Lung Cancer with Brain Metastasis Receiving Tyrosine Kinase Inhibitors. Transl. Oncol..

[142] Grossman R., Haim O., Abramov S., Shofty B., Artzi M. (2021). Differentiating Small-Cell Lung Cancer from Non-Small-Cell Lung Cancer Brain Metastases Based on MRI Using Efficientnet and Transfer Learning Approach. Technol. Cancer Res. Treat..

[143] Li Y., Lv X., Chen C., Yu R., Wang B., Wang D., Hou D. (2024). A Deep Learning Model Integrating Multisequence MRI to Predict EGFR Mutation Subtype in Brain Metastases from Non-Small Cell Lung Cancer. Eur. Radiol. Exp..

[144] Liao C.Y., Lee C.C., Yang H.C., Chen C.J., Chung W.Y., Wu H.M., Guo W.Y., Liu R.S., Lu C.F. (2023). Predicting Survival after Radiosurgery in Patients with Lung Cancer Brain Metastases Using Deep Learning of Radiomics and EGFR Status. Phys. Eng. Sci. Med..

[145] Jünger S.T., Hoyer U.C.I., Schaufler D., Laukamp K.R., Goertz L., Thiele F., Grunz J.P., Schlamann M., Perkuhn M., Kabbasch C. (2021). Fully Automated MR Detection and Segmentation of Brain Metastases in Non-Small Cell Lung Cancer Using Deep Learning. J. Magn. Reson. Imaging.

[146] Tulum G. (2023). Novel Radiomic Features versus Deep Learning: Differentiating Brain Metastases from Pathological Lung Cancer Types in Small Datasets. Br. J. Radiol..

[147] Sui L., Chang S., Xue L., Wang J., Zhang Y., Yang K., Gao B.-L., Yin X. (2023). Deep Learning Based on Enhanced MRI T1 Imaging to Differentiate Small-Cell and Non-Small-Cell Primary Lung Cancers in Patients with Brain Metastases. Curr. Med. Imaging.

[148] Haim O., Abramov S., Shofty B., Fanizzi C., DiMeco F., Avisdris N., Ram Z., Artzi M., Grossman R. (2022). Predicting EGFR Mutation Status by a Deep Learning Approach in Patients with Non-Small Cell Lung Cancer Brain Metastases. J. Neurooncol..

[149] Ishimoto Y., Ide S., Watanabe K., Oyu K., Kasai S., Umemura Y., Sasaki M., Nagaya H., Tatsuo S., Nozaki A. (2024). Usefulness of Pituitary High-Resolution 3D MRI with Deep-Learning-Based Reconstruction for Perioperative Evaluation of Pituitary Adenomas. Neuroradiology.

[150] Wang H., Zhang W., Li S., Fan Y., Feng M., Wang R. (2021). Development and Evaluation of Deep Learning-Based Automated Segmentation of Pituitary Adenoma in Clinical Task. J. Clin. Endocrinol. Metab..

[151] Yan X., Lin B., Fu J., Li S., Wang H., Fan W., Fan Y., Feng M., Wang R., Fan J. (2023). Deep-Learning-Based Automatic Segmentation and Classification for Craniopharyngiomas. Front. Oncol..

[152] Zhu H., Fang Q., Huang Y., Xu K. (2020). Semi-Supervised Method for Image Texture Classification of Pituitary Tumors via CycleGAN and Optimized Feature Extraction. BMC Med. Inform. Decis. Mak..

[153] Zhu L., Zhang L., Hu W., Chen H., Li H., Wei S., Chen X., Ma X. (2022). A Multi-Task Two-Path Deep Learning System for Predicting the Invasiveness of Craniopharyngioma. Comput. Methods Programs Biomed..

[154] Park H., Nam Y.K., Kim H.S., Park J.E., Lee D.H., Lee J., Kim S., Kim Y.H. (2023). Deep Learning-Based Image Reconstruction Improves Radiologic Evaluation of Pituitary Axis and Cavernous Sinus Invasion in Pituitary Adenoma. Eur. J. Radiol..

[155] Fang Y., Wang H., Cao D., Cai S., Qian C., Feng M., Zhang W., Cao L., Chen H., Wei L. (2024). Multi-Center Application of a Convolutional Neural Network for Preoperative Detection of Cavernous Sinus Invasion in Pituitary Adenomas. Neuroradiology.

[156] Staartjes V.E., Serra C., Muscas G., Maldaner N., Akeret K., van Niftrik C.H.B., Fierstra J., Holzmann D., Regli L. (2018). Utility of Deep Neural Networks in Predicting Gross-Total Resection after Transsphenoidal Surgery for Pituitary Adenoma: A Pilot Study. Neurosurg. Focus.

[157] Sato M., Tateishi K., Murata H., Kin T., Suenaga J., Takase H., Yoneyama T., Nishii T., Tateishi U., Yamamoto T. (2018). Three-Dimensional Multimodality Fusion Imaging as an Educational and Planning Tool for Deep-Seated Meningiomas. Br. J. Neurosurg..

[158] Jun Y., Park Y.W., Shin H., Shin Y., Lee J.R., Han K., Ahn S.S., Lim S.M., Hwang D., Lee S.K. (2023). Intelligent Noninvasive Meningioma Grading with a Fully Automatic Segmentation Using Interpretable Multiparametric Deep Learning. Eur. Radiol..

[159] Chen J., Xue Y., Ren L., Lv K., Du P., Cheng H., Sun S., Hua L., Xie Q., Wu R. (2023). Predicting Meningioma Grades and Pathologic Marker Expression via Deep Learning. Eur. Radiol..

[160] Azamat S., Buz-Yalug B., Dindar S.S., Yilmaz Tan K., Ozcan A., Can O., Ersen Danyeli A., Pamir M.N., Dincer A., Ozduman K. (2024). Susceptibility-Weighted MRI for Predicting NF-2 Mutations and S100 Protein Expression in Meningiomas. Diagnostics.

[161] She Z., Marzullo A., Destito M., Spadea M.F., Leone R., Anzalone N., Steffanoni S., Erbella F., Ferreri A.J.M., Ferrigno G. (2023). Deep Learning-Based Overall Survival Prediction Model in Patients with Rare Cancer: A Case Study for Primary Central Nervous System Lymphoma. Int. J. Comput. Assist. Radiol. Surg..

[162] Quon J.L., Bala W., Chen L.C., Wright J., Kim L.H., Han M., Shpanskaya K., Lee E.H., Tong E., Iv M. (2020). Deep Learning for Pediatric Posterior Fossa Tumor Detection and Classification: A Multi-Institutional Study. AJNR Am. J. Neuroradiol..

[163] Cheng D., Zhuo Z., Du J., Weng J., Zhang C., Duan Y., Sun T., Wu M., Guo M., Hua T. (2024). A Fully Automated Deep-Learning Model for Predicting the Molecular Subtypes of Posterior Fossa Ependymomas Using T2-Weighted Images. Clin. Cancer Res..

[164] Kujawa A., Dorent R., Connor S., Oviedova A., Okasha M., Grishchuk D., Ourselin S., Paddick I., Kitchen N., Vercauteren T. (2022). Automated Koos Classification of Vestibular Schwannoma. Front. Radiol..

[165] Lee C.-C., Lee W.K., Wu C.C., Lu C.F., Yang H.C., Chen Y.W., Chung W.Y., Hu Y.S., Wu H.M., Wu Y.T. (2021). Applying Artificial Intelligence to Longitudinal Imaging Analysis of Vestibular Schwannoma Following Radiosurgery. Sci. Rep..

[166] Yu Y., Song G., Zhao Y., Liang J., Liu Q. (2023). Prediction of Vestibular Schwannoma Surgical Outcome Using Deep Neural Network. World Neurosurg..

[167] Lee W.K., Wu C.C., Lee C.C., Lu C.F., Yang H.C., Huang T.H., Lin C.Y., Chung W.Y., Wang P.S., Wu H.M. (2020). Combining Analysis of Multi-Parametric MR Images into a Convolutional Neural Network: Precise Target Delineation for Vestibular Schwannoma Treatment Planning. Artif. Intell. Med..

[168] Jayachandran Preetha C., Meredig H., Brugnara G., Mahmutoglu M.A., Foltyn M., Isensee F., Kessler T., Pflüger I., Schell M., Neuberger U. (2021). Deep-Learning-Based Synthesis of Post-Contrast T1-Weighted MRI for Tumour Response Assessment in Neuro-Oncology: A Multicentre, Retrospective Cohort Study. Lancet. Digit. Health.

[169] Tang S., Liao J., Long Y. (2019). Comparative Assessment of the Efficacy of Gross Total versus Subtotal Total Resection in Patients with Glioma: A Meta-Analysis. Int. J. Surg..

[170] Hoshide R., Jandial R. (2016). Human Cerebral Cortex Map 2.0. Neurosurgery.

[171] Rajdeo P., Aronow B., Surya Prasath V.B. (2024). Deep Learning-Based Multimodal Spatial Transcriptomics Analysis for Cancer. Adv. Cancer Res..

[172] Brown T.J., Brennan M.C., Li M., Church E.W., Brandmeir N.J., Rakszawski K.L., Patel A.S., Rizk E.B., Suki D., Sawaya R. (2016). Association of the Extent of Resection with Survival in Glioblastoma: A Systematic Review and Meta-Analysis. JAMA Oncol..

[173] Bjorland L.S., Mahesparan R., Fluge Ø., Gilje B., Kurz K.D., Farbu E. (2023). Impact of Extent of Resection on Outcome from Glioblastoma Using the RANO Resect Group Classification System: A Retrospective, Population-Based Cohort Study. Neuro-Oncol. Adv..

[174] Huang J., Shlobin N.A., Decuypere M., Lam S.K. (2022). Deep Learning for Outcome Prediction in Neurosurgery: A Systematic Review of Design, Reporting, and Reproducibility. Neurosurgery.

[175] Ho D.J., Agaram N.P., Jean M.H., Suser S.D., Chu C., Vanderbilt C.M., Meyers P.A., Wexler L.H., Healey J.H., Fuchs T.J. (2023). Deep Learning–Based Objective and Reproducible Osteosarcoma Chemotherapy Response Assessment and Outcome Prediction. Am. J. Pathol..

[176] Obata Y., Parkinson D.Y., Pelt D.M., Acevedo C. (2025). Enhancing Synchrotron Radiation Micro-CT Images Using Deep Learning: An Application of Noise2Inverse on Bone Imaging. J. Synchrotron Radiat..

[177] Minnema J., Wolff J., Koivisto J., Lucka F., Batenburg K.J., Forouzanfar T., van Eijnatten M. (2021). Comparison of Convolutional Neural Network Training Strategies for Cone-Beam CT Image Segmentation. Comput. Methods Programs Biomed..

[178] Patel A., More B., Rege I., Ranade D. (2024). Clinical Diagnosis and Management of Multiple Cerebral Ring-Enhancing Lesions-Study of 50 Patients at a Tertiary Healthcare Center. J. Cancer Res. Ther..

[179] Peker E., Ünal S., Uludağ S.B., Zorlu N.S.Y. (2024). Ring-Enhancing Lesions-Differentiation with MRI. Br. J. Hosp. Med..

[180] Waqas A., Tripathi A., Ramachandran R.P., Stewart P., Rasool G. (2023). Multimodal Data Integration for Oncology in the Era of Deep Neural Networks: A Review. Front. Artif. Intell..

[181] Boehringer A.S., Sanaat A., Arabi H., Zaidi H. (2023). An Active Learning Approach to Train a Deep Learning Algorithm for Tumor Segmentation from Brain MR Images. Insights Imaging.

[182] Zhang Z., Li J., Tian C., Zhong Z., Jiao Z., Gao X. (2021). Quality-Driven Deep Active Learning Method for 3D Brain MRI Segmentation. Neurocomputing.

[183] Liu X., Shih H.A., Xing F., Santarnecchi E., El Fakhri G., Woo J. (2023). Incremental Learning for Heterogeneous Structure Segmentation in Brain Tumor MRI. Medical Image Computing and Computer Assisted Intervention—MICCAI 2023.

[184] Li R., Ye J., Huang Y., Jin W., Xu P., Guo L. (2023). A Continuous Learning Approach to Brain Tumor Segmentation: Integrating Multi-Scale Spatial Distillation and Pseudo-Labeling Strategies. Front. Oncol..

[185] Kordnoori S., Sabeti M., Shakoor M.H., Moradi E. (2024). Deep Multi-Task Learning Structure for Segmentation and Classification of Supratentorial Brain Tumors in MR Images. Interdiscip. Neurosurg. Adv. Tech. Case Manag..

[186] Huang H., Yang G., Zhang W., Xu X., Yang W., Jiang W., Lai X. (2021). A Deep Multi-Task Learning Framework for Brain Tumor Segmentation. Front. Oncol..

[187] Ronneberger O., Fischer P., Brox T. (2015). U-Net: Convolutional Networks for Biomedical Image Segmentation. Lect. Notes Comput. Sci..

[188] Zhou Z., Rahman Siddiquee M.M., Tajbakhsh N., Liang J. (2018). Unet++: A Nested u-Net Architecture for Medical Image Segmentation. Deep Learning in Medical Image Analysis and Multimodal Learning for Clinical Decision Support.

[189] Milletari F., Navab N., Ahmadi S.A. V-Net: Fully Convolutional Neural Networks for Volumetric Medical Image Segmentation. Proceedings of the 2016 Fourth International Conference on 3D Vision (3DV).

[190] Huang S.Y., Hsu W.L., Hsu R.J., Liu D.W. (2022). Fully Convolutional Network for the Semantic Segmentation of Medical Images: A Survey. Diagnostics.

[191] Vedpathak S., Soni P., Gaikwad S., Parmar M. 2D Brain MRI Segmentation: U-Nets Versus Optimized DeepLab Models. Proceedings of the 2024 IEEE International Conference on Information Technology, Electronics and Intelligent Communication Systems (ICITEICS).

[192] Yamanakkanavar N., Choi J.Y., Lee B. (2022). SM-SegNet: A Lightweight Squeeze M-SegNet for Tissue Segmentation in Brain MRI Scans. Sensors.

[193] Chen J., Mei J., Li X., Lu Y., Yu Q., Wei Q., Luo X., Xie Y., Adeli E., Wang Y. (2024). TransUNet: Rethinking the U-Net Architecture Design for Medical Image Segmentation through the Lens of Transformers. Med. Image Anal..

[194] Wang W., Chen C., Ding M., Yu H., Zha S., Li J. (2021). TransBTS: Multimodal Brain Tumor Segmentation Using Transformer. Lect. Notes Comput. Sci..

[195] Hatamizadeh A., Tang Y., Nath V., Yang D., Myronenko A., Landman B., Roth H.R., Xu D. UNETR: Transformers for 3D Medical Image Segmentation. Proceedings of the 2022 IEEE/CVF Winter Conference on Applications of Computer Vision (WACV).

[196] Amri Y., Ben Slama A., Mbarki Z., Selmi R., Trabelsi H. (2025). Automatic Glioma Segmentation Based on Efficient U-Net Model Using MRI Images. Intell. Med..

[197] Gi Y., Oh G., Jo Y., Lim H., Ko Y., Hong J., Lee E., Park S., Kwak T., Kim S. (2024). Study of Multistep Dense U-Net-Based Automatic Segmentation for Head MRI Scans. Med. Phys..

[198] Shaheema S.B., Suganya Devi K., Muppalaneni N.B. (2024). Explainability Based Panoptic Brain Tumor Segmentation Using a Hybrid PA-NET with GCNN-ResNet50. Biomed. Signal Process. Control.

[199] Hossain S., Chakrabarty A., Gadekallu T.R., Alazab M., Piran M.J. (2024). Vision Transformers, Ensemble Model, and Transfer Learning Leveraging Explainable AI for Brain Tumor Detection and Classification. IEEE J. Biomed. Health Inform..

[200] Mertes S., Huber T., Weitz K., Heimerl A., André E. (2022). GANterfactual—Counterfactual Explanations for Medical Non-Experts Using Generative Adversarial Learning. Front. Artif. Intell..

[201] Singla S., Eslami M., Pollack B., Wallace S., Batmanghelich K. (2023). Explaining the Black-Box Smoothly—A Counterfactual Approach. Med. Image Anal..

[202] Wang P., Yang Q., He Z., Yuan Y. (2023). Vision Transformers in Multi-Modal Brain Tumor MRI Segmentation: A Review. Meta-Radiology.

[203] Wang J., Lu S.Y., Wang S.H., Zhang Y.D. (2024). RanMerFormer: Randomized Vision Transformer with Token Merging for Brain Tumor Classification. Neurocomputing.

[204] Jiang J., Chen X., Tian G., Liu Y. ViG-UNet: Vision Graph Neural Networks for Medical Image Segmentation. Proceedings of the 2023 IEEE 20th International Symposium on Biomedical Imaging (ISBI).

[205] Puzio T., Matera K., Karwowski J., Piwnik J., Białkowski S., Podyma M., Dunikowski K., Siger M., Stasiołek M., Grzelak P. (2025). Deep Learning-Based Automatic Segmentation of Brain Structures on MRI: A Test-Retest Reproducibility Analysis. Comput. Struct. Biotechnol. J..

